# Polypharmacy and deprescribing: challenging the old and embracing the new

**DOI:** 10.1186/s12877-022-03408-6

**Published:** 2022-09-07

**Authors:** Lisa Kouladjian O’Donnell, Kinda Ibrahim

**Affiliations:** 1 Departments of Clinical Pharmacology and Ageing, Kolling Institute, Royal North Shore Hospital, Faculty of Medicine and Health, The University of Sydney, NSW St Leonards, Australia; 2 Academic Geriatric Medicine, Faculty of Medicine, University Hospital Southampton, University of Southampton, Southampton, UK; 3 Applied Research Collaboration Wessex, The National Institute of Health and Care Research (NIHR), University of Southampton, Southampton, UK

People are living longer, and the World Health Organisation predicts that by 2050, 22% of the world’s population will be aged 60 years or older [1]. A common consequence of ageing is multimorbidity, which can lead to the use of multiple medications by an individual, also known as polypharmacy. The appropriateness of polypharmacy has been contested in various settings and population groups, and there is growing concern that many older people use an inappropriately high number of medications, with varying degrees of medication complexity [2]. In a study that aimed to determine the optimal discriminating number of medications associated with poor outcomes in community-dwelling older adults, the use of five or more medications estimated medication-related adverse events such as frailty, mortality and falls [3]. For many years, this definition of polypharmacy has been used in clinical trials for recruiting older adults and for estimating the effect of interventions to reduce polypharmacy.

However, with the changing landscape of older adults, is defining polypharmacy as five or more medications within research still appropriate? A study that investigated the evidence for an optimal cut-point to define polypharmacy in long-term care facilities to predict poor outcomes in older adults concluded that there was no single definition of polypharmacy that was predictive of all adverse outcomes, however, the common definition of “9 or more regular medications” was the average at predicting fall-related hospitalisation, falls and mortality [4]. Additionally, The Quality Indicator Program that is implemented within Australian Commonwealth-funded residential aged care facilities, defines polypharmacy as the prescription of 9 or more medications to an aged care resident, reflecting the clinical application of monitoring for polypharmacy within this setting [5]. This also leads us to question whether the definition of polypharmacy is “setting-dependent”—does the definition of polypharmacy shift as the older person transitions through different levels of care?

Should the focus of research be on appropriateness of medications instead of number of medications? It is estimated that more than 50% of older people are prescribed at least one medication that causes more harm than benefit [6]. Inappropriate polypharmacy is associated with significant poor health outcomes such as injurious falls, cognitive impairment, hospitalization, and death, and can lead to wasteful healthcare expenditure [7]. Challenging current concepts and definitions of polypharmacy can lead to newer ways of understanding its impact, which can inform the design of interventions to address polypharmacy.

Deprescribing defined as “the process of withdrawing medications, under the supervision of a healthcare practitioner, which may no longer be of benefit or may be causing harm”, is considered the solution to polypharmacy, and is highlighted as a driver to addressing this major public-health issue worldwide [8]. For example in the UK, the recent report published by the Department of Health and Social Care highlighted the problem of overprescribing and suggested a reduction in the volume of prescription items in primary care of 10% through planned structured medication review and deprescribing interventions [9]. In Australia, Quality Use of Medicines and Medicines Safety was announced as Australia's 10th National Health Priority by the Council of Australian Governments (COAG) Health Council in November 2019, with subsequent funding calls for research and innovation in optimising medicine use. Deprescribing efforts are increasing globally, reinforced by the formation of regional and national deprescribing networks such as those that exist in Australia (Australian Deprescribing Network, ADeN), Canada (Canadian Deprescribing Network, CaDeN), Europe (European Deprescribing Network, EDeN and Network of European Researchers in Deprescribing, NERD), and the United States (US Deprescribing Research Network, USDeN), aimed at bringing multidisciplinary groups of professionals together to share resources, research, successes, and challenges [10].

Deprescribing has been shown to be feasible and can be performed safely in older people [11], yet it can be difficult to initiate and sustain, and infrequently performed even when supported by decision-making tools [12]. Studies have identified that shared decision-making, gradual introduction of the topic, clear communication with the patient and working as part of a multidisciplinary team can facilitate deprescribing, whilst consultation constraints, patients' fears of negative consequences and difficulty understanding the terminology and information provided around deprescribing are perceived as barriers to deprescribing [13, 14]. However, there is still a lack of a common understanding of what are the best practices or approaches for implementing deprescribing interventions in different cultures, healthcare systems, and clinical settings. For example, there is a paucity of studies investigating appropriate prescribing and deprescribing interventions for older Indigenous Australians [15]. This is particularly important, as deprescribing requires complex changes to established patterns of behaviour at the individual, organisational, and systems levels. It is crucial to understand how deprescribing works, and for whom, how to sustain its implementation in clinical practice, and the role of technology in assisting implementation [16].

Recently, some studies suggested that medication review/deprescribing interventions work best in combination with other interventions. For example, recent reviews have shown that deprescribing antihypertensives in older people and taking non-pharmacological approaches, such as regular exercise and reduced dietary sodium, is beneficial in reducing risk of falls [17, 18]. There is growing evidence that a multidisciplinary approach to deprescribing could have potential advantages on staff workload, and patients’ clinical outcomes [19]. However, more research is needed in this area to explain the roles and responsibilities of different stakeholders involved in the care of older people in the deprescribing process. Innovative models of deprescribing involving different stakeholders (such as advanced nurse practitioners and social prescribers) and the cost effectiveness of these interventions should be a target for future research. Furthermore, research should also focus on how deprescribing could be sustained and become part of practitioners’ routine practice to enable cultural change in relation to prescribing habits and behaviours.

Deprescribing interventions have been criticised to date for their lack of focus on measuring clinical outcomes and concentrating on process outcomes (feasibility and acceptability) and their success in reducing inappropriate medications and polypharmacy. This is why many systematic reviews have concluded that deprescribing is feasible and can reduce medication use, however, there is less evidence on the impact of deprescribing on clinical and person-centred outcomes [20]. A 2017 review of 47 deprescribing interventional studies among older people reported the outcome measures that were most commonly used were number of medications used (35%), healthcare services use (23%), and adverse events (21%) and very few reported clinical or patients-reported outcomes (7%) [21]. In 2020 Aubert et al. reviewed the outcome measures used in 93 deprescribing intervention studies and reported that 97% used at least one measure related to appropriate prescribing, and only 34% used patient-reported measures (outcomes, preferences, and experiences) [22]. Rankin et al. proposed a set of 16 different core outcomes for trials aimed at improving the appropriateness of polypharmacy in older people, and identified the 7 highest-ranked outcomes: serious adverse drug reactions, medication appropriateness, falls, medication regimen complexity, quality of life, mortality, and medication side effects [23]. The impact of deprescribing on some geriatric syndromes such as frailty and sarcopenia status have rarely been reported [11]. Considering the strong relationship between polypharmacy and frailty and the potential to reverse frailty status in animal studies [24, 25], it is important to understand the impact of deprescribing on frailty in older adult populations. Furthermore, more safety data is needed to increase uptake of deprescribing in clinical practice, by addressing the barrier of fear of consequences of stopping medications, and to enhance including deprescribing in national and international guidelines and policies. Safety has been defined in terms of reported adverse drug withdrawal events (ADWEs), return of medical condition(s), hospital admission and/or all-cause mortality [11, 26]. The absence of change in health status following deprescribing, can be perceived as positive, because it could mean less treatment burden, lower medication costs and low carbon footprint prescribing [27]. However, research is needed to identify the safety concerns of deprescribing and large randomized controlled trials are needed to understand the prevalence and severity of ADWEs from deprescribing and the best strategies to reduce their risk [26].

Studies have suggested that for deprescribing to succeed, it requires effective communication that resonates with patients and caregivers [26, 28]. How clinicians communicate about deprescribing may affect to what extent patients and caregivers understand and involve themselves with the process. When patients are fully informed about the risks and benefits of available treatments and are engaged in decision-making, they are more likely to accept deprescribing recommendations [28]. In order to increase engagement of patients and caregivers in deprescribing conversations and facilitate shared-decision making, multiple tools (including fact sheets and leaflets but also materials such as decision aids and option grids) have been developed [29]. However, many important limitations to these tools have been identified, including their above average reading level, unbalanced information of the potential benefits and harms of deprescribing, and lack of elicitation of patients’ treatment goals and preferences. Further research is needed to understand how best to engage and communicate information about deprescribing to patients/caregivers taking into account factors such as health literacy and cognitive abilities. Co-designing materials with patients and carers may engage and incorporate their personalised needs and concerns, given the mounting evidence that patient-centred care and shared-decision making can improve patient satisfaction, adherence, quality of life and overall health outcomes [30].

The aim of this collection is to progress the field of polypharmacy and deprescribing research, by publishing studies addressing the key priorities that are highlighted in this editorial. We hope this freely accessible collection will empower and drive ground-breaking research in polypharmacy and deprescribing to improve quality use of medicines and outcomes in older adults, globally.

## References

[1] Ageing and Health,the World Health Organisation. https://www.who.int/news-room/fact-sheets/detail/ageing-and-health

[2] Wastesson JW, Morin L, Tan ECK, Johnell K (2018). An update on the clinical consequences of polypharmacy in older adults: a narrative review. Expert Opin Drug Saf.

[3] Gnjidic D, Hilmer SN, Blyth FM, Naganathan V, Waite L, Seibel MJ, McLachlan AJ, Cumming RG, Handelsman DJ, Le Couteur DG (2012). Polypharmacy cutoff and outcomes: five or more medicines were used to identify community-dwelling older men at risk of different adverse outcomes. J Clin Epidemiol.

[4] Wang KN, Tan ECK, Ilomäki J, Gilmartin-Thomas JFM, Sluggett JK, Cooper T, Robson L, Bell JS (2021). What is the best definition of polypharmacy for predicting falls, hospitalizations, and mortality in long-term care facilities?. J Am Med Dir Assoc.

[5] Aged Care Quality Bulletin #36 – December 2021. [https://www.agedcarequality.gov.au/news-centre/newsletter/quality-bulletin-36-december-2021]

[6] Hillen JB, Soulsby N, Clarke M (2021). Too many pills, too many sick older Australians: Working together is key. Aust J Gen Pract.

[7] Maher RL, Hanlon J, Hajjar ER (2014). Clinical consequences of polypharmacy in elderly. Expert Opin Drug Saf.

[8] Reeve E, Gnjidic D, Long J, Hilmer S (2015). A systematic review of the emerging definition of 'deprescribing' with network analysis: implications for future research and clinical practice. Br J Clin Pharmacol.

[9] Good for you, good for us, good for everybody. [https://assets.publishing.service.gov.uk/government/uploads/system/uploads/attachment_data/file/1019475/good-for-you-good-for-us-good-for-everybody.pdf ]

[10] Thompson AR, Kim CS, Kim GE, Keller MS, Marcum ZA, Brandt NJ (2022). Challenges and successes of global deprescribing networks: a qualitative key informant study. J Gerontol Nurs.

[11] Ibrahim K, Cox NJ, Stevenson JM, Lim S, Fraser SDS, Roberts HC (2021). A systematic review of the evidence for deprescribing interventions among older people living with frailty. BMC Geriatr.

[12] Kouladjian O'Donnell L, Gnjidic D, Sawan M, Reeve E, Kelly PJ, Chen TF, Bell JS, Hilmer SN. Impact of the Goal-directed Medication Review Electronic Decision Support System on Drug Burden Index: A cluster-randomised clinical trial in primary care. Br J Clin Pharmacol. 2021;87:1499–511. 10.1111/bcp.14557 32960464

[13] Peat G, Fylan B, Marques I, Raynor DK, Breen L, Olaniyan J, Alldred DP (2022). Barriers and facilitators of successful deprescribing as described by older patients living with frailty, their informal carers and clinicians: a qualitative interview study. BMJ Open.

[14] Turner JP, Tannenbaum C (2017). Older Adults' Awareness of Deprescribing: A Population-Based Survey. J Am Geriatr Soc.

[15] Moulds RF, McDermott DR (2019). Improving prescribing for Indigenous Australians. Med J Aust.

[16] Wu H, Kouladjian O'Donnell L, Fujita K, Masnoon N, Hilmer SN (2021). Deprescribing in the Older Patient: A Narrative Review of Challenges and Solutions. Int J Gen Med.

[17] Benetos A, Petrovic M, Strandberg T (2019). Hypertension Management in Older and Frail Older Patients. Circ Res.

[18] Kazeminia M, Daneshkhah A, Jalali R, Vaisi-Raygani A, Salari N, Mohammadi M (2020). The effect of exercise on the older adult’s blood pressure suffering hypertension: systematic review and meta-analysis on clinical trial studies. Int J Hypertens.

[19] Fixen DR, Farro SA, Shanbhag P, Parnes BL, Vejar MV (2022). Multidisciplinary approach to deprescribing sedative-hypnotic medications in geriatric primary care. J Prim Care Community Health.

[20] Lundby C, Pottegård A (2022). Considerations regarding choice of primary outcome in clinical trials in deprescribing. Br J Clin Pharmacol.

[21] Beuscart JB, Pont LG, Thevelin S, Boland B, Dalleur O, Rutjes AWS, Westbrook JI, Spinewine A (2017). A systematic review of the outcomes reported in trials of medication review in older patients: the need for a core outcome set. Br J Clin Pharmacol.

[22] Aubert CE, Kerr EA, Maratt JK, Klamerus ML, Hofer TP (2020). Outcome measures for interventions to reduce inappropriate chronic drugs: a narrative review. J Am Geriatr Soc.

[23] Rankin A, Cadogan CA, In Ryan C, Clyne B, Smith SM, Hughes CM (2018). Core outcome set for trials aimed at improving the appropriateness of polypharmacy in older people in primary care. J Am Geriatr Soc.

[24] Ng TP, Feng L, Nyunt MS, Feng L, Niti M, Tan BY, Chan G, Khoo SA, Chan SM, Yap P (2015). Nutritional, physical, cognitive, and combination interventions and frailty reversal among older adults: a randomized controlled trial. Am J Med.

[25] Mach J, Gemikonakli G, Logan C, Vander Wyk B, Allore H, Ekambareshwar S, Kane AE, Howlett SE, de Cabo R, Le Couteur DG, et al. Chronic polypharmacy with increasing Drug Burden Index (DBI) exacerbates frailty and impairs physical function, with effects attenuated by deprescribing, in aged mice. J Gerontol A Biol Sci Med Sci. 2020. 10.1093/gerona/glaa060 PMC8140051 32147704

[26] Reeve E, Moriarty F, Nahas R, Turner JP, Kouladjian O'Donnell L, Hilmer SN (2018). A narrative review of the safety concerns of deprescribing in older adults and strategies to mitigate potential harms. Expert Opin Drug Saf.

[27] Cussans A, Harvey G, Kemple T, Tomson M (2021). Interventions to Reduce the Environmental Impact of Medicines: A UK perspective✰. J Climate Change Health.

[28] Green AR, Aschmann H, Boyd CM, Schoenborn N (2021). Assessment of Patient-Preferred Language to Achieve Goal-Aligned Deprescribing in Older Adults. JAMA Netw Open.

[29] Fajardo MA, Weir KR, Bonner C, Gnjidic D, Jansen J (2019). Availability and readability of patient education materials for deprescribing: An environmental scan. Br J Clin Pharmacol.

[30] Reeve E, Shakib S, Hendrix I, Roberts MS, Wiese MD (2014). Review of deprescribing processes and development of an evidence-based, patient-centred deprescribing process. Br J Clin Pharmacol.

