# Correction to: Polypharmacy and deprescribing: Challenging the old and embracing the new

**DOI:** 10.1186/s12877-022-03465-x

**Published:** 2022-10-03

**Authors:** Lisa Kouladjian O’Donnell, Kinda Ibrahim

**Affiliations:** 1 Departments of Clinical Pharmacology and Ageing, Kolling Institute, Royal North Shore Hospital, Faculty of Medicine and Health, The University of Sydney, St Leonards, NSW Australia; 2 Academic Geriatric Medicine, Faculty of Medicine, University Hospital Southampton, University of Southampton, Southampton, UK; 3 Applied Research Collaboration Wessex, The National Institute of Health and Care Research (NIHR), University of Southampton, Southampton, UK

## Correction to: *BMC Geriatr***22**, 734 (2022). 10.1186/s12877-022-03408-6

Following publication of the original article [1], it was reported that there was a typographical error in the reference to the English Deprescribing Network, EdEN. This was erroneously referred to as the European Desprescribing Network.

The original article [1] has been updated.
